# Use of National and International Growth Charts for Studying Height in European Children: Development of Up-To-Date European Height-For-Age Charts

**DOI:** 10.1371/journal.pone.0042506

**Published:** 2012-08-15

**Authors:** Marjolein Bonthuis, Karlijn J. van Stralen, Enrico Verrina, Alberto Edefonti, Elena A. Molchanova, Anita C. S. Hokken-Koelega, Franz Schaefer, Kitty J. Jager

**Affiliations:** 1 European Society for Pediatric Nephrology/European Renal Association - European Dialysis and Transplant Association Registry, Department of Medical Informatics, Academic Medical Center, University of Amsterdam, Amsterdam, The Netherlands; 2 Gaslini Children’s Hospital, Genoa, Italy; 3 Fondazione Ca’ Granda IRCCS Ospedale Maggiore Policlinico, Milano, Italy; 4 Department of Kidney Transplantation, Russian Children’s Clinical Hospital, Moscow, Russia; 5 Department of Paediatrics, Subdivision of Endocrinology, Erasmus University Medical Center/Sophia Children’s Hospital, Rotterdam, The Netherlands; 6 University Children’s Hospital, Heidelberg, Germany; University of South Australia, Australia

## Abstract

**Background:**

Growth charts based on data collected in different populations and time periods are key tools to assess children’s linear growth. We analyzed the impact of geographic factors and the secular trend on height-for-age charts currently used in European populations, developed up-to-date European growth charts, and studied the effect of using different charts in a sample of growth retarded children.

**Methods and Findings:**

In an international survey we obtained 18 unique national height-for-age charts from 28 European countries and compared them with charts from the World Health Organization (WHO), Euro-Growth reference, and Centers of Disease Control and Prevention (CDC). As an example, we obtained height data from 3,534 children with end-stage renal disease (ESRD) from 13 countries via the ESPN/ERA-EDTA registry, a patient group generally suffering from growth retardation. National growth charts showed a clear secular trend in height (mean height increased on average 0.6 cm/decade) and a North-South height gradient in Europe. For countries without a recent (>1990) national growth chart novel European growth charts were constructed from Northern and Southern European reference populations, reflecting geographic height differences in mean final height of 3.9 cm in boys and 3.8 cm in girls. Mean height SDS of 2- to 17-year-old ESRD patients calculated from recent national or derived European growth charts (−1.91, 95% CI: −1.97 to −1.85) was significantly lower than when using CDC or WHO growth charts (−1.55, 95% CI: −1.61 to −1.49) (*P*<0.0001).

**Conclusion:**

Differences between height-for-age charts may reflect true population differences, but are also strongly affected by the secular trend in height. The choice of reference charts substantially affects the clinical decision whether a child is considered short-for-age. Therefore, we advocate using recent national or European height-for-age charts derived from recent national data when monitoring growth of healthy and diseased European children.

## Introduction

Age- and sex-specific growth charts are essential clinical tools to monitor the adequacy of children’s longitudinal growth.[1]–[3] Impaired growth is a major global public health issue [4], and its correct diagnosis is crucial to prompt timely intervention. Although many different national and international growth charts for height exist [1], national growth charts are unavailable in numerous countries. Therefore, the 1977 National Center for Health Statistics/World Health Organization (NCHS/WHO) references have been recommended for worldwide use. [3] Recently, the Centers of Disease Control and Prevention (CDC) [5] and the WHO [6] released revised versions of the NCHS/WHO growth charts. However, as both datasets are mainly based on data collected more than forty years ago, they may be outdated because of the secular trend in height. Moreover, the NCHS/WHO charts describe the growth of US children, and thus do not represent an international sample. Therefore, in 2006 the WHO released international growth standards for children aged 0–5 years based on growth data of children from six countries around the globe. The growth data were collected from children living under optimal conditions.[7]–[9] These growth standards were intended to replace national growth charts in young children. Furthermore, in 2000 the Euro-Growth Study Group released reference charts for infants younger than 3 years based on a sample from twelve European countries. [10], [11] Although the WHO growth standards were designed to be applicable to all children around the globe, in some Northern European countries heights of 0–5 year olds appeared to be larger than the supposedly ‘ideal heights’ according to WHO growth standards [12]–[14], questioning the universal applicability of the WHO growth standards. In daily practice, most countries preferentially apply their national height-for-age charts [1] whenever available, even when these are based on ‘outdated’ data. While this practice may cause problems because of the secular trend in height, the use of the CDC data may not provide a sufficient solution. [15].

**Table 1:** Characteristics of different growth charts.

Country or growth chart	Years of Survey	Ages (years)	Number of children	Sample representativefor entire country	Exclusion criteria	Modeling technique used
Belgium [19]	2002–2004	0–21	15,989	No^1^	Non Belgian parents, chronic illness,born before 37 weeks	LMS
Czech Republic [20]	2001	0–19	59,000	Yes		
Denmark [21]	1974	0–18	13,210	Yes	Non Danish parents	
Estonia [22]	1996–1997	2–20	20,367	Yes		cubic splines
Finland [23], [24]	1959–1983	0–19	2,897	No^1^	Born before 36 weeks, birth weight<2500 g, chronic illness	Spline function
France [25]	1953–1975	0–20	497			Weighted LS
Greece [26]	2000–2001	0–18	9,797	No^1^		LMS
Germany [13]	2003–2006	0–18	17,079	Yes	Illness and medications that could affect growth	LMS
Hungary [27]	1979	3–18	5,685			
Italy [28]	1996–2004	2–20	69,917	Yes		Extended mechanistic growth function (EMGF)^2^
Lithuania [29]	1996–2003	0–18	9,000	Yes		
Netherlands [30]	1996–1997	0–25	14,500	Yes	Illness and medications that could affect growth	LMS
Norway [18]	2003–2006	0–19	8,299	No	One/both parents outside Northern Europe, chronic illness, prematurity	LMS
Russia [31] 3	1980’s	0–17				
Spain [32]	2000–2004	0–18	32,064	Yes	Non Spanish parents, chronic illness, medication use	LMS
Sweden [33]	1992	0–18	3,650	Yes		Polynomial function
Switzerland [34]	1954–1976	0–20	274	Yes	Non Swiss parents, birth weight <2500 g, illness	Spline function
United Kingdom[35], [65] 4	1972–1993	0–23	25,385	Yes	Non-Caucasian children	LMS
CDC [37]	1963–1994	0–19	950,928	Yes	Birth weight <1500 g	LMS
WHO growth standards [9]	1996–2003	0–5	8,440	Yes	Health, environmental, or economic constraints to growth (morbidities, multiple birth etc.)	BCPE with cubic splines
WHO growth charts [6]	1963–1974	5–19	22,917	Yes	Birth weight <1500 g	
Euro-Growth reference [10]	1990–1996	0–3	2,245	Yes	Illness, birth before 37 weeks, birth weight <2500 g	

1 Although the sample was not population based, the authors stated that height of sampled children will likely not be different from children living in other regions in the country;

2 Method similar to LMS method;

3 Russian charts are published in a key pediatric book, and are commonly applied by pediatricians throughout Russia;

4 The UK-WHO growth charts are applied in clinical practice in the United Kingdom and constitute growth data from WHO growth standards with birth data from the British 1990 charts. As the WHO growth standards are already included in the analyses, the new WHO-UK growth charts were not considered.

The impact of the choice of growth charts is even more important to consider when analyzing longitudinal growth of children from different countries, a task of increasing importance with the emergence of multinational pediatric registries and clinical trials. We recently encountered this problem when studying longitudinal growth in European children with end-stage renal disease within the framework of the ESPN/ERA-EDTA Registry. Highly diverse populations, major regional variation in the tempo of growth, and the availability of several national growth charts of variable actuality in Europe illustrate the challenge of applying adequate reference methods to a heterogeneous population.[7], [12], [13], [16]–[18] In this study, we addressed this issue in a three-stage approach: (1) assess the appropriateness of different growth charts for height in current use in European countries; (2) develop an optimized set of valid height-for-age charts applicable throughout Europe; and (3) illustrate the impact of the use of different height-for-age charts on an international population with growth retardation, i.e. children with end-stage renal disease.

**Figure 1:**
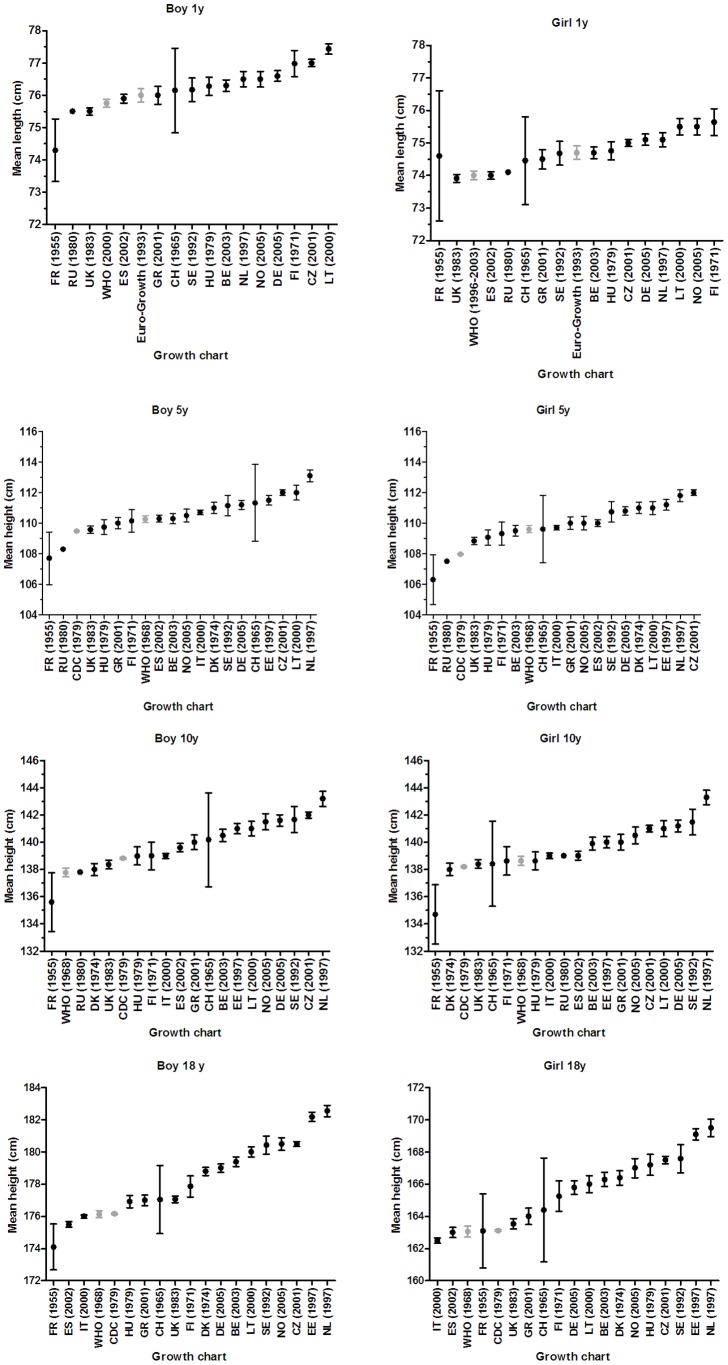
Mean ±2 **SE of height for different growth charts by sex and age.**

## Methods

### Growth Charts

#### Currently available height-for-age charts

In November 2010, we conducted a survey among pediatric centers in 32 European countries in order to obtain information on height-for-age charts used in clinical practice. A response was obtained from 28 countries (88%). Eighteen countries used a unique height-for-age chart based on data from their own country. Table 1 provides an overview of the different charts included in this study. National growth charts for height were available for Belgium [19], Czech Republic [20], Denmark [21], Estonia [22], Finland [23], [24], France [25], Greece [26], Germany [13], Hungary [27], Italy [28], Lithuania [29], the Netherlands [30], Norway [18], Russia [31], Spain [32], Sweden [33], Switzerland [34], and the United Kingdom. [35], [36] Furthermore, we included the international reference charts for height of Euro-Growth [10], CDC [37] and WHO. [6], [9].

**Figure 2:**
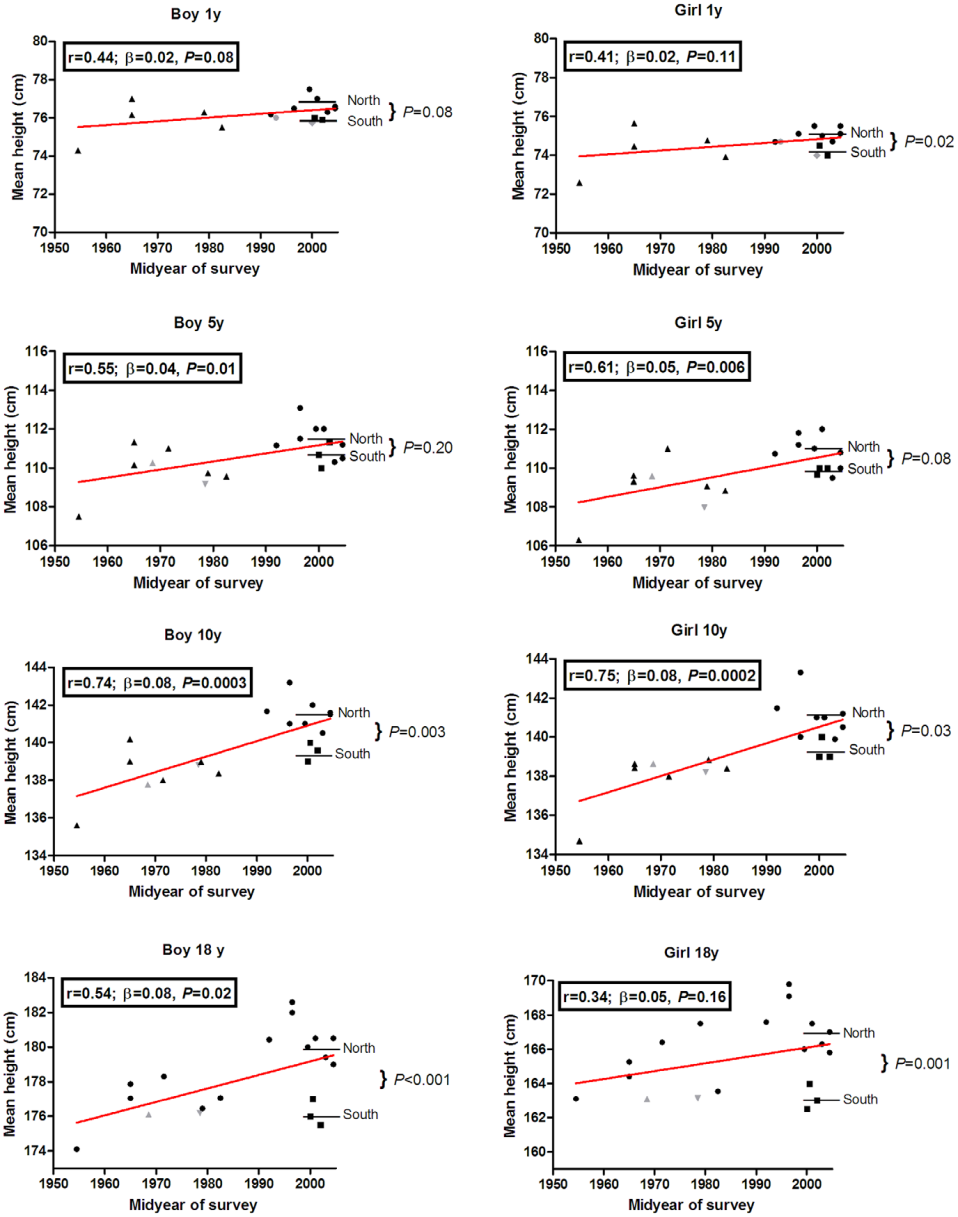
Mean heights by midyear of data collection for different growth charts. Mean heights are shown for 1, 5, 10, and 18 year old boys and girls (▴ = national growth charts before 1990; ▪ = national growth charts of Southern European countries after 1990; • = national growth charts for Northern European countries after 1990; ▾ = CDC growth charts for 5, 10, and 18 year olds; ▴ = WHO growth charts for 5, 10, and 18 year olds; • = Euro-Growth for one year olds; and ♦ = WHO growth charts for one-year olds; horizontal lines represent the mean height for Northern and Southern Europe in the growth charts based on data after 1990).

The Euro-Growth reference included longitudinal growth data up to 36 months of age from a sample of twelve European countries. The WHO growth standards consist of longitudinal growth measurements from birth to 24 months, followed by cross-sectional data till the age of 5 years based on data from six countries around the globe.

**Figure 3:**
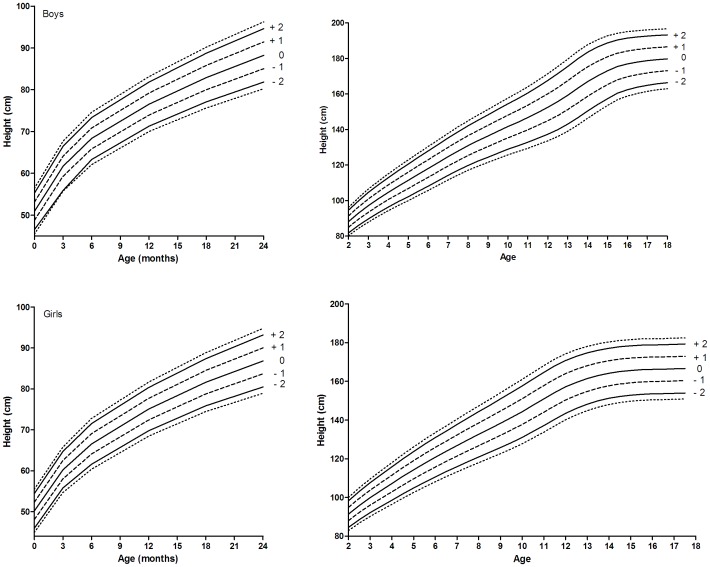
New growth charts proposed for Northern European countries without recent national growth charts. Outer lines indicate −2.5 SDS and +2.5 SDS.

#### Construction of growth charts for Northern and Southern Europe

To replace older national height-for-age charts and to provide reference tables for those countries in which national growth charts are unavailable, we developed two sets of European reference charts for height accounting for the secular trend as well as for the North-South gradient in height. [16] Based on serial t-testing of different cut-offs to identify the partition yielding the most significant difference in mean heights, we considered charts for which the midyear of height data collection was 1990 as up-to-date. For each recent national chart we weighted the mean height and the standard deviation for each six month age interval for the country’s total population size in 2010 and combined these values to create a weighted average for Northern and Southern Europe.

**Figure 4:**
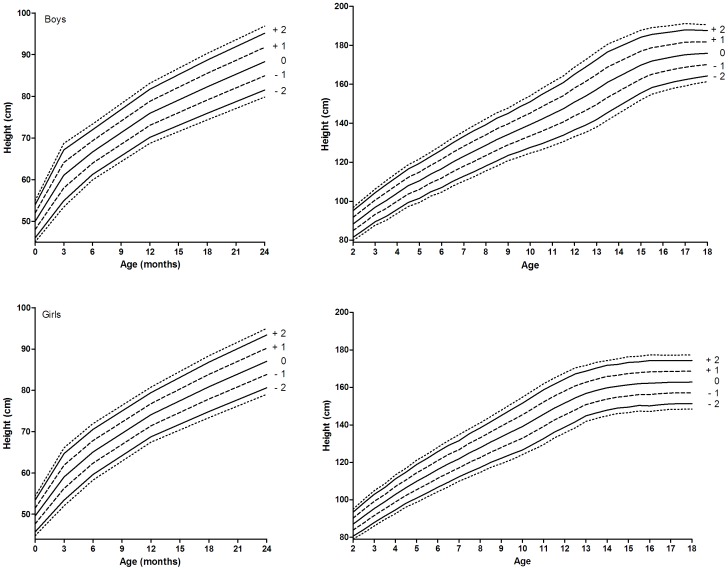
New growth charts proposed for Southern European countries without recent national growth charts. Outer lines indicate −2.5 SDS and +2.5 SDS.

### Pediatric End-Stage Renal Disease Patients: an Example

#### Subjects

Data on pediatric end-stage renal disease patients were collected within the framework of the ESPN/ERA-EDTA Registry and included date of birth, sex, treatment modality (i.e. dialysis or transplantation) at start of renal replacement therapy, and changes in treatment modality. For the present study, we included patients younger than 18 years, who started renal replacement therapy during the period 1995–2010. The most recent height measurement available in the registry was included for: Belgium, Czech Republic, Finland, France, Italy (2–17 years old), Lithuania, Norway, Romania, Russia, Slovakia, Spain, Switzerland, and the United Kingdom.

**Table 2:** Mean length SDS for 0–1 year old end-stage renal disease patients.

Country		Mean length SDS (SE)				
	N	National^1^		Recent National/European	WHO	Euro-Growth^2^
Belgium	2	−2.62 (1.64)	−2.62 (1.64)		−3.07 (1.19)	−2.62 (1.59)
Czech Republic	3	−1.80 (0.70)	−1.80 (0.70)		−1.91 (0.91)	−1.99 (0.85)
Finland	7	−1.39 (0.46)	−1.33 (0.47)		−1.24 (0.50)	−1.26 (0.49)
France	20	−1.91 (0.42)	−2.12 (0.36)		−2.56 (0.43)	−2.46 (0.41)
Lithuania	2	−1.00 (0.26)	−1.00 (0.26)		−1.07 (0.36)	−1.11 (0.36)
Norway	1	−0.90	−0.90		−0.83	−1.03
Romania	1	1.97	1.16		1.25	1.08
Russia	4	−2.50 (1.15)	−3.23 (1.13)		−3.22 (1.26)	−3.27 (1.22)
Spain	22	−1.72 (0.33)	−1.72 (0.33)		−1.99 (0.35)	−1.99 (0.38)
Switzerland	3	−0.89 (0.86)	−0.75 (0.85)		−1.09 (0.61)	−0.86 (0.76)
United Kingdom	21	−3.13 (0.37)	−2.83 (0.28)		−2.84 (0.30)	−2.87 (0.30)
All countries^3^	86	−2.04 (0.19)	−2.05 (0.17)		−2.24 (0.19)	−2.22 (0.19)

1 National growth charts refer to both growth charts based on data collected before 1990 as well as to recent national growth charts;

2 As the CDC recommends the use of the WHO growth standards for children under the age of 2 years, mean length SDS values based on the CDC for children younger than 2 years are not reported;

3 These values represent the average length SDS of children with ESRD from all European countries together.

#### Data analysis

The most recent height measurement of each patient was converted to length/height-for-age standard deviation scores (SDS) using recent national growth charts whenever available. For countries lacking recent national charts SD scores were calculated using the European height-for-age charts constructed in this study. The mean SD scores for patients from separate European countries as well as for all countries combined were then compared with the length SDS calculated from WHO growth standards and Euro-Growth reference for 0–1 year olds and with height SDS according to CDC and WHO growth charts for 2–17 year old children. CDC growth charts in the 0–1 year old group were not included in the comparison since for this age group the use of the WHO growth standards is recommended. [38].

**Table 3:** Mean height SDS for 2–17 year old end-stage renal disease patients.

Country		Mean height SDS (SE)			
	N	National^1^	Recent National/European	WHO	CDC^2^
Belgium	8	−0.16 (0.47)	−0.16 (0.47)	0.01 (0.49)	0.04 (0.47)
Czech Republic	45	−1.81 (0.17)	−1.81 (0.17)	−1.23 (0.16)	−1.21 (0.16)
Finland	124	−1.46 (0.11)	−1.79 (0.11)	−1.25 (0.11)	−1.23 (0.11)
France	289	−1.47 (0.11)	−1.83 (0.11)	−1.59 (0.10)	−1.59 (0.10)
Italy	541	−2.13 (0.07)	−2.13 (0.07)	−1.83 (0.06)	−1.80 (0.06)
Lithuania	28	−1.52 (0.40)	−1.52 (0.40)	−1.12 (0.40)	−1.10 (0.38)
Norway	54	−1.82 (0.19)	−1.82 (0.19)	−1.30 (0.17)	−1.28 (0.17)
Romania	73	−3.39 (0.21)	−3.68 (0.20)	−3.22 (0.18)	−3.22 (0.17)
Russia	234	−2.06 (0.13)	−2.74 (0.12)	−2.23 (0.12)	−2.21 (0.12)
Slovakia	6	−1.57 (0.47)	−1.54 (0.45)	−1.13 (0.46)	−1.07 (0.45)
Spain	729	−1.43 (0.05)	−1.43 (0.05)	−1.35 (0.05)	−1.36 (0.05)
Switzerland	170	−1.25 (0.11)	−1.26 (0.12)	−1.06 (0.10)	−1.05 (0.10)
United Kingdom	1101	−1.79 (0.03)	−1.99 (0.02)	−1.43 (0.02)	−1.44 (0.02)
All countries^3^	3402	−1.75 (0.03)	−1.91 (0.03)	−1.55 (0.03)	−1.55 (0.03)

1 National growth charts refer to both growth charts based on data collected before 1990 as well as to recent national growth charts;

2 Height-for-age reference values according to the Euro-Growth reference are not available for children over the age of 3 years;

3 These values represent the average height SDS of children with ESRD from all European countries together.

To determine whether children were short-for-age we used a length/height SDS cut-off value of <−2.

**Figure 5:**
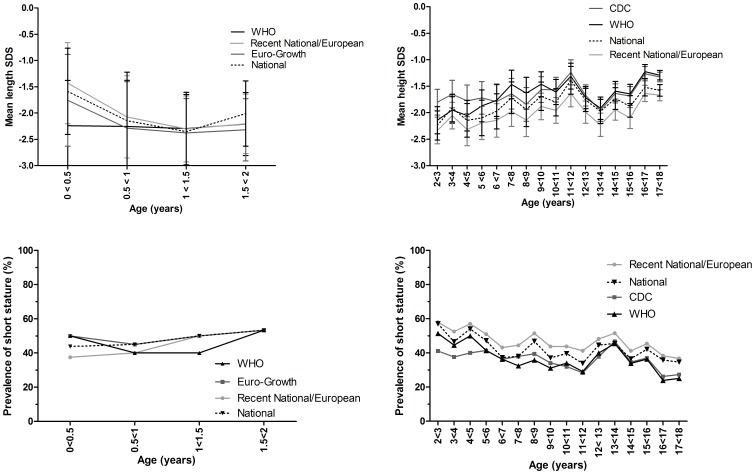
Height-SDS and prevalence of short stature for different growth charts in end-stage renal disease patients. Mean height-SDS and prevalence of short stature according to different growths are shown separately for 0–1 year old and 2–17 year old patients. The National growth charts include both recent national growth charts as the growth charts based on data collected before 1990.

All statistical analyses were carried out using SAS version 9.2 (SAS Institute Inc., Cary, NC, USA).

## Results

### Differences between Growth Charts

The height distribution differed substantially between growth charts. Figure 1 shows the absolute mean heights ±2 SE according to different reference charts (i.e. charts based on recent data as well as those based on data collected before 1990) by sex and for four selected ages.

Most national height-for-age charts showed higher mean heights than those according to the Euro-Growth reference and the WHO growth standards for one-year-old children, or according to CDC and WHO growth charts for 5, 10 and 18 year old children. Mean height differences among 1 year old children were only marginal and not significant.

We tested whether these differences in mean heights were related to the secular trend in height by plotting the midyear of the survey in which growth chart height data were collected against the mean height for the four selected age groups (Figure 2). Recent surveys showed consistently higher mean heights, reflecting a positive secular trend in height. In 18 year old girls the secular trend was, however, smaller compared to younger ages. Differences in mean heights were highly significant when collection midyear of 1990 was used as a cut-off to determine which charts were up-to-date. As a result, we considered growth charts based on data collected before 1990 to be outdated.

Besides the secular trend in height, growth charts also varied due to differences in study design (in-and exclusion criteria) and due to modeling techniques used to construct the charts (Table 1). Between the recent growth charts for height (>1990) there were, however, no apparent differences between these factors.

### Growth Charts for Height for Northern and Southern Europe

The height-for-age chart for Northern Europe was based on the charts from Belgium, [19] Czech Republic [20], Estonia [22], Germany [13], Lithuania [29], the Netherlands [30], Norway [18], and Sweden [33], whereas the height-for-age chart for Southern Europe was compiled from the charts of Greece [26], Italy [28], and Spain. [32] Recent height-for-age charts for Southern European countries generally showed lower mean heights than recent charts for Northern European countries, suggesting a North-South gradient in height (Figure 2), especially in children older than 5 years of age where a significant gradient was observed. For example, 18- year-old boys in Southern Europe were on average 3.9 cm shorter than their peers living in Northern European countries (*P*<0.05). To allow for this geographical height gradient and to correct for the secular trend we developed two separate height-for-age charts for Northern and Southern European children (Figures 3 and 4). Standard deviation scores (SDS) by sex at 6-month age intervals are given in Appendix S1.

### Differences in Height-for-age Charts in Pediatric End-stage Renal Disease Patients

To illustrate the impact of the use of different growth charts on linear growth data from a population frequently suffering from growth retardation, we applied the charts to a population of children with end-stage renal disease. For countries with national height-for-age charts based on data collected before 1990, mean height SDS is shown according to these national growth charts, as well as to our newly constructed European growth charts to illustrate the differences. The European growth charts were used in children from those countries in which recent national growth charts for height are unavailable, i.e. the Northern European charts in children from Finland, Russia, Slovakia, and the United Kingdom, and the Southern European charts in children from France, Romania, and Switzerland.

Length/height SDS calculated using different growth charts for 0–1 year old and 2–17 year old end-stage renal disease patients are shown in Table 2 and 3, respectively. For 0–1 year old end-stage renal disease patients we found small, non-significant, differences when applying different growth charts. The mean length SDS according to the Euro-Growth reference (−2.22±0.19) and the WHO growth standards (−2.24±0.19) were slightly lower than for national growth charts: −2.04±0.19 for national charts including growth charts from before the 1990s and −2.05±0.17 for recent national/European growth charts. The proportion of children rated short-for-age was similar according to Euro-Growth reference (50%), WHO growth standards (47%), national growth charts (including those from before 1990) (49%) and recent national/European growth charts (47%) (Fig. 5).

For 2–17 year old end-stage renal disease patients mean height SDS based on recent national/European growth charts were lower compared to the national growth charts including those based on data collected before 1990 (Table 3). Overall, the mean height SDS according to recent national/European growth charts (−1.91±0.03) was significantly lower than when calculated using the CDC and WHO growth charts (−1.55±0.03), while the height SDS calculated according to national growth charts including growth charts from before 1990 yielded intermediate values (−1.75±0.03) (Table 3). Hence, children appeared shorter according to recent national/European growth charts (Fig. 5) and more children were classified as short-for-age according to these recent national/European growth charts (44%) as compared to CDC (34%) or WHO (33%) and national growth charts including charts from before 1990 (40%).

## Discussion

We found that the mean heights of the general pediatric population, as reflected in growth charts, vary substantially. To determine whether the longitudinal growth pattern of a child is normal, height should be compared to an appropriate reference population. [16], [39], [40] Defining the appropriate reference population is, however, a matter of debate. Some studies suggested the use of one single height-for-age chart worldwide [7], [16], whereas other studies found significant population differences in height, therefore, advocating the use of national height-for-age charts. [12], [13], [18].

The variation in linear growth charts appeared to be related both to the era of data collection and to true population differences. Growth charts for height constructed from data collected before 1990, including the CDC and WHO growth charts, yielded generally lower mean heights than those developed more recently. Since 1850 there has been a positive secular trend in height among European populations. [15], [41], [42] Like in the United States [5], in many Northern European countries as well as in Italy this secular trend slowed down or even reached a plateau since the 1980s/1990s [15], [43]–[46], whereas in other countries, like Belgium, Spain, and Portugal, average heights might still increase. [45].

We found considerable differences in mean heights among European populations, with children from Northern Europe generally being taller than those from Southern Europe [43], [45]–[47], suggesting a North-South height gradient in European children older than 5 years of age. For example, in the European growth charts developed in this study mean height at age 18 years is 166.7 cm for Northern European girls while it is 162.8 cm for Southern European girls. These findings are in keeping with previous studies reporting a clear difference in height between Scandinavian countries, the Netherlands and Germany as compared to countries in the Mediterranean region (e.g. France, Italy, Portugal, and Spain). [43], [45], [47] These marked population differences could be related to environmental, socio-economical, and/or genetic factors. [16], [47] Population differences could also be related to differences in the extent of the secular trend, which started earlier in Northern Europe than in Southern Europe, leading to taller statures in Northern Europe. [41] Furthermore, as the onset of puberty occurs later in Northern Europe, possibly due to a lower obesity prevalence [48] or genetic factors [49], final adult height could be higher in Northern Europe than in Southern Europe. [49] Some of the length variation among infants might have been caused by differences in the ages at which measurements switched from recumbent length to standing height, as well as by the difficulty to measure recumbent length. [50] It has been suggested that weight-for-age might provide a more reliable tool for infant growth monitoring. [51].

The secular trend in height mandates regular updating of growth references in order to detect children who have a short stature relative to their peers. Recent single-country height-for-age charts based on sufficiently large representative samples of children can be assumed to provide optimal reference information. However, such studies are infrequently performed due to high workload, high costs and limited scientific value. [52] In our study, national growth charts for height were lacking in 10 out of 28 countries (36%), outdated charts were used in 5 others (18%), and national charts from other countries, which were not necessarily the most appropriate ones, were used in 3 countries (11%). The selection of children included in national growth charts or the use of modeling techniques did not differ systematically between recent national growth studies from Northern and Southern Europe. Therefore, the reference charts developed in this study for Northern and Southern European populations based on all recent national height-for-age charts available to us, may take the role to monitor linear growth of children in countries where recent national growth charts are lacking. It should be emphasized though that this ‘geographic interpolation’ approach cannot replace and should not preclude regular monitoring of the growth and nutritional status of (healthy) children in all European countries.

The issue raised here is not only of anthropological interest, but has also important clinical implications. Since clinical decision-making such as the indication for growth hormone or other growth promoting therapies [53], [54] are based on the comparison of an individual child’s height to the height distribution of the peer population, differences between height-for-age charts may have considerable implications for individual patients. Also, for public health purposes, as stunting is one of the main contributors to the global burden of disease [4], it is of major importance to prompt intervention in the right children. When a reference population is taller, more children will meet the criterion of having a height SDS below −2. So, based on older national, CDC, or WHO reference charts (5–19 years) fewer children will be considered eligible for growth promoting therapy compared to recent national growth charts. This was shown in Australia, where children having a height SDS < −2.3 (or <1^st^ percentile) based on CDC growth charts were considered eligible for subsidized rhGH treatment. Theoretically, 1% of the Australian children should meet this criterion. However, only 0.5% of the Australian children had a height SDS below the 1^st^ percentile of the CDC growth charts. [53] We found that 34% of the 2–17 year old children with end-stage renal disease would be eligible for growth hormone therapy by using WHO or CDC growth charts, whereas 44% would meet the criterion when using recent national or the European growth charts derived here. Regulatory authorities both at the national and at the European level have precisely defined the indications for growth-modulating therapies according to height and growth velocity criteria. [55], [56] It is of major concern that in many instances these well-defined criteria are applied using inappropriate reference charts. The variable actuality and representativeness of height-for-age charts used in different European countries violates the objective of equal access to health care for all European children [57], and defines the need for a Europe-wide, periodically updated study of growth, development and nutrition in healthy children.

Finally, in international growth or registry studies, when comparing linear growth data from different ethnic or geographic sources, international charts do not represent local growth appropriately due to population differences in height. Nevertheless, in these studies, the newly constructed height-for-age charts for Northern and Southern European populations might serve as appropriate reference charts because these better approach geographical height differences compared to one single international growth chart (e.g. CDC or WHO growth chart).

A possible limitation of our study is that the overview of national growth charts in this study may be incomplete, as we only included those charts which are applied in pediatric nephrology practice, a discipline in which growth retardation is relatively common. The choice of national growth charts for studying height could be slightly different for other medical disciplines. Moreover, the European height-for-age charts were not smoothed using the standard LMS method [58] as we were not able to retrieve the original data from the included growth studies. However, the original growth data were smoothed prior to construction of the European growth charts which largely removed random variation in the original data, resulting in relatively smooth charts.

As the original height-for-age charts on which our European charts are based did not provide information on gestational age and neither on linear growth of children from non-Western immigrants, our growth charts might not be applicable to small for gestational age babies or children from ethnic minorities living in Europe. However, several reference charts correcting for gestational age [59], [60], as well as reference charts specifically designed for growth monitoring of immigrant children [61]–[64], are available in literature. Furthermore, our charts also will require periodical updating in order to keep pace with height and ethnic changes in the reference population.

### Conclusion

We found considerable differences in mean heights among different growth charts with height SDS in children differing depending on the reference chart used. The differences are likely due to the secular trend in height as well as to geographical differences in height. Therefore, we developed new height-for-age charts for Northern and Southern European countries. When monitoring longitudinal growth of European children, we propose to use recent national growth charts. However, if these are lacking we suggest that height-for-age charts for Northern and Southern Europe based on recent national charts, like ours, are preferable to other national or international height-for-age charts.

## Supporting Information

Appendix S1 A) Growth charts constructed for Northern European boys; B) Growth charts constructed for Northern European girls; C) Growth charts constructed for Southern European boys; D) Growth Charts constructed for Southern European girls.
(DOC)
