# Supplementary material for: Use of National and International Growth Charts for Studying Height in European Children: Development of Up-To-Date European Height-For-Age Charts
Source: PLoS One. 2012 Aug 15;7(8):e42506. doi: 10.1371/journal.pone.0042506 (PMC3419735; doi:10.1371/journal.pone.0042506)
Supplement: Appendix S1 — A) Growth charts constructed for Northern European boys; B) Growth charts constructed for Northern European girls; C) Growth charts constructed for Southern European boys; D) Growth Charts constructed for Southern European girls. (DOC) [file pone.0042506.s001.doc]

**Appendix S1**

**A**

| **Age (years)** | **SD** | **-2.5** | **-2** | **-1** | **0** | **+1** | **+2** | **+2.5** |
| --- | --- | --- | --- | --- | --- | --- | --- | --- |
| **0** | 2.18 | 45.47 | 46.57 | 48.75 | 50.93 | 53.12 | 55.30 | 56.39 |
| **0.25** | 2.39 | 55.74 | 55.93 | 59.32 | 61.72 | 64.11 | 66.50 | 67.69 |
| **0.5** | 2.51 | 62.08 | 63.34 | 65.85 | 68.36 | 70.87 | 73.37 | 74.63 |
| **1** | 2.63 | 70.01 | 71.32 | 73.95 | 76.58 | 79.21 | 81.85 | 83.16 |
| **1.5** | 2.91 | 75.64 | 77.10 | 80.00 | 82.91 | 85.82 | 88.73 | 90.18 |
| **2** | 3.20 | 80.25 | 81.85 | 85.05 | 88.24 | 91.44 | 94.64 | 96.24 |
| **2.5** | 3.45 | 84.33 | 86.05 | 89.51 | 92.96 | 96.41 | 99.87 | 101.59 |
| **3** | 3.74 | 87.85 | 89.72 | 93.46 | 97.20 | 100.94 | 104.68 | 106.56 |
| **3.5** | 3.93 | 91.26 | 93.23 | 97.16 | 101.09 | 105.03 | 108.96 | 110.93 |
| **4** | 4.15 | 94.34 | 96.42 | 100.60 | 104.72 | 108.87 | 113.02 | 115.10 |
| **4.5** | 4.36 | 97.23 | 99.41 | 103.77 | 108.13 | 112.48 | 116.84 | 119.02 |
| **5** | 4.60 | 99.92 | 102.22 | 106.81 | 111.41 | 116.01 | 120.61 | 122.91 |
| **5.5** | 4.76 | 102.92 | 105.30 | 110.06 | 114.82 | 119.58 | 124.34 | 126.72 |
| **6** | 4.95 | 105.70 | 108.18 | 113.12 | 118.07 | 123.01 | 127.96 | 130.43 |
| **6.5** | 5.09 | 108.69 | 111.24 | 116.33 | 121.42 | 126.51 | 131.60 | 134.15 |
| **7** | 5.25 | 111.61 | 114.24 | 119.48 | 124.73 | 129.98 | 135.23 | 137.85 |
| **7.5** | 5.41 | 114.40 | 117.10 | 122.52 | 127.93 | 133.35 | 138.76 | 141.50 |
| **8** | 5.57 | 117.04 | 119.82 | 125.39 | 130.96 | 136.54 | 142.11 | 144.89 |
| **8.5** | 5.76 | 119.34 | 122.22 | 127.97 | 133.73 | 139.49 | 145.25 | 148.13 |
| **9** | 5.96 | 121.51 | 124.48 | 130.44 | 136.40 | 142.36 | 148.31 | 151.29 |
| **9.5** | 6.17 | 123.64 | 126.73 | 132.90 | 139.08 | 145.25 | 151.42 | 154.51 |
| **10** | 6.39 | 125.74 | 128.93 | 135.32 | 141.72 | 148.11 | 154.50 | 157.70 |
| **10.5** | 6.64 | 127.56 | 130.88 | 137.52 | 144.16 | 150.80 | 157.44 | 160.76 |
| **11** | 6.93 | 129.43 | 132.90 | 139.83 | 146.76 | 153.69 | 160.62 | 164.08 |
| **11.5** | 7.22 | 131.53 | 135.15 | 142.37 | 149.60 | 156.82 | 164.04 | 167.66 |
| **12** | 7.56 | 133.60 | 137.39 | 144.95 | 152.51 | 160.08 | 167.64 | 171.43 |
| **12.5** | 7.88 | 136.01 | 139.95 | 147.83 | 155.71 | 163.58 | 171.46 | 175.40 |
| **13** | 8.11 | 139.02 | 143.07 | 151.18 | 159.28 | 167.39 | 175.50 | 179.55 |
| **13.5** | 8.31 | 142.44 | 146.59 | 154.90 | 163.20 | 171.51 | 179.82 | 183.97 |
| **14** | 8.28 | 146.31 | 150.45 | 158.73 | 167.01 | 175.30 | 183.58 | 187.72 |
| **14.5** | 8.11 | 149.98 | 154.04 | 162.15 | 170.27 | 178.38 | 186.50 | 190.55 |
| **15** | 7.85 | 153.50 | 157.42 | 165.27 | 173.12 | 180.96 | 188.81 | 192.74 |
| **15.5** | 7.48 | 156.78 | 160.52 | 168.00 | 175.47 | 182.95 | 190.43 | 194.17 |
| **16** | 7.26 | 158.81 | 162.43 | 169.69 | 176.95 | 184.21 | 191.47 | 195.09 |
| **16.5** | 7.07 | 160.30 | 163.84 | 170.90 | 177.97 | 185.04 | 192.11 | 195.64 |
| **17** | 6.93 | 161.45 | 164.91 | 171.84 | 178.77 | 185.70 | 192.63 | 196.09 |
| **17.5** | 6.81 | 162.39 | 165.80 | 172.60 | 179.41 | 186.22 | 193.03 | 196.43 |
| **18** | 6.73 | 163.00 | 166.36 | 173.09 | 179.81 | 186.54 | 193.27 | 196.63 |

**B**

| **Age (years)** | **SD** | **-2.5** | **-2** | **-1** | **0** | **+1** | **+2** | **+2.5** |
| --- | --- | --- | --- | --- | --- | --- | --- | --- |
| **0** | 2.11 | 44.93 | 45.98 | 48.10 | 50.21 | 52.33 | 54.44 | 55.50 |
| **0.25** | 2.33 | 54.68 | 55.80 | 58.03 | 60.26 | 62.50 | 64.73 | 65.80 |
| **0.5** | 2.48 | 60.37 | 61.61 | 64.09 | 66.57 | 69.05 | 71.53 | 72.77 |
| **1** | 2.64 | 68.46 | 69.78 | 72.42 | 75.06 | 77.69 | 80.33 | 81.65 |
| **1.5** | 2.87 | 74.46 | 75.89 | 78.77 | 81.64 | 84.51 | 87.38 | 88.82 |
| **2** | 3.17 | 78.91 | 80.50 | 83.67 | 86.84 | 90.02 | 93.19 | 94.77 |
| **2.5** | 3.42 | 82.91 | 84.62 | 88.04 | 91.47 | 94.89 | 98.31 | 100.03 |
| **3** | 3.64 | 86.80 | 88.62 | 92.26 | 95.90 | 99.54 | 103.18 | 105.00 |
| **3.5** | 3.87 | 90.26 | 92.19 | 96.06 | 99.93 | 103.80 | 107.67 | 109.61 |
| **4** | 4.08 | 93.40 | 95.44 | 99.52 | 103.60 | 107.69 | 111.77 | 113.81 |
| **4.5** | 4.31 | 96.44 | 98.60 | 102.91 | 107.22 | 111.53 | 115.84 | 117.99 |
| **5** | 4.52 | 99.58 | 101.84 | 106.36 | 110.89 | 115.41 | 119.93 | 122.19 |
| **5.5** | 4.70 | 102.57 | 104.93 | 109.63 | 114.33 | 119.04 | 123.74 | 126.10 |
| **6** | 4.91 | 105.37 | 107.82 | 112.73 | 117.64 | 122.55 | 127.46 | 129.91 |
| **6.5** | 5.08 | 108.14 | 110.68 | 115.75 | 120.83 | 125.90 | 130.98 | 133.52 |
| **7** | 5.23 | 110.78 | 113.40 | 118.62 | 123.85 | 129.07 | 134.30 | 136.91 |
| **7.5** | 5.46 | 113.19 | 115.92 | 121.38 | 126.84 | 132.31 | 137.77 | 140.50 |
| **8** | 5.66 | 115.64 | 118.47 | 124.13 | 129.79 | 135.45 | 141.11 | 143.94 |
| **8.5** | 5.87 | 117.97 | 120.91 | 126.78 | 132.64 | 138.51 | 144.38 | 147.31 |
| **9** | 6.07 | 120.31 | 123.34 | 129.41 | 135.48 | 141.54 | 147.61 | 150.64 |
| **9.5** | 6.29 | 122.65 | 125.79 | 132.08 | 138.37 | 144.65 | 150.94 | 154.09 |
| **10** | 6.50 | 125.07 | 128.32 | 134.82 | 141.31 | 147.81 | 154.30 | 157.55 |
| **10.5** | 6.65 | 127.72 | 131.05 | 137.70 | 144.36 | 151.01 | 157.67 | 161.00 |
| **11** | 6.78 | 130.73 | 134.12 | 140.90 | 147.68 | 154.45 | 161.23 | 164.62 |
| **11.5** | 6.87 | 133.75 | 137.19 | 144.06 | 150.93 | 157.81 | 164.68 | 168.12 |
| **12** | 6.90 | 137.00 | 140.45 | 147.36 | 154.26 | 161.16 | 168.07 | 171.52 |
| **12.5** | 6.82 | 140.12 | 143.53 | 150.36 | 157.18 | 164.00 | 170.82 | 174.23 |
| **13** | 6.74 | 142.70 | 146.07 | 152.81 | 159.55 | 166.28 | 173.02 | 176.39 |
| **13.5** | 6.64 | 144.87 | 148.19 | 154.83 | 161.47 | 168.11 | 174.76 | 178.08 |
| **14** | 6.54 | 146.70 | 149.97 | 156.51 | 163.04 | 169.58 | 176.12 | 179.39 |
| **14.5** | 6.45 | 148.07 | 151.30 | 157.75 | 164.21 | 170.66 | 177.11 | 180.34 |
| **15** | 6.43 | 149.00 | 152.21 | 158.64 | 165.07 | 171.50 | 177.93 | 181.15 |
| **15.5** | 6.37 | 149.71 | 152.90 | 159.27 | 165.65 | 172.02 | 178.39 | 181.58 |
| **16** | 6.35 | 150.18 | 153.35 | 159.70 | 166.05 | 172.41 | 178.76 | 181.93 |
| **16.5** | 6.32 | 150.42 | 153.58 | 159.90 | 166.21 | 172.53 | 178.85 | 182.01 |
| **17** | 6.31 | 150.54 | 153.69 | 160.00 | 166.31 | 172.62 | 178.92 | 182.08 |
| **17.5** | 6.32 | 150.69 | 153.85 | 160.17 | 166.49 | 172.81 | 179.13 | 182.29 |
| **18** | 6.32 | 150.86 | 154.02 | 160.33 | 166.65 | 172.96 | 179.28 | 182.43 |

C

| **Age (years)** | **SD** | **-2.5** | **-2** | **-1** | **0** | **+1** | **+2** | **+2.5** |
| --- | --- | --- | --- | --- | --- | --- | --- | --- |
| **0** | 1.99 | 45.04 | 46.03 | 48.01 | 50.00 | 51.99 | 53.97 | 54.96 |
| **0.25** | 3.05 | 53.44 | 54.96 | 58.01 | 61.06 | 64.11 | 67.16 | 68.69 |
| **0.5** | 2.68 | 59.91 | 61.25 | 63.93 | 66.61 | 69.29 | 71.97 | 73.31 |
| **1** | 2.88 | 68.71 | 70.15 | 73.03 | 75.92 | 78.80 | 81.69 | 83.13 |
| **1.5** | 3.19 | 74.32 | 75.91 | 79.11 | 82.30 | 85.50 | 88.69 | 90.29 |
| **2** | 3.40 | 79.81 | 81.51 | 84.91 | 88.31 | 91.72 | 95.12 | 96.82 |
| **2.5** | 3.58 | 83.57 | 85.36 | 88.94 | 92.52 | 96.10 | 99.68 | 101.47 |
| **3** | 3.70 | 87.65 | 89.50 | 93.20 | 96.90 | 100.61 | 104.31 | 106.16 |
| **3.5** | 4.08 | 90.17 | 92.21 | 96.28 | 100.36 | 104.43 | 108.51 | 110.55 |
| **4** | 4.22 | 91.76 | 93.87 | 98.09 | 102.32 | 106.54 | 110.76 | 112.88 |
| **4.5** | 4.29 | 97.26 | 99.41 | 103.70 | 107.99 | 112.28 | 116.57 | 118.71 |
| **5** | 4.48 | 99.29 | 101.52 | 106.00 | 110.48 | 114.95 | 119.43 | 121.67 |
| **5.5** | 4.52 | 102.55 | 104.81 | 109.33 | 113.85 | 118.37 | 122.89 | 125.14 |
| **6** | 4.82 | 104.58 | 106.99 | 111.81 | 116.62 | 121.44 | 126.26 | 128.67 |
| **6.5** | 4.94 | 107.69 | 110.17 | 115.11 | 120.05 | 125.00 | 129.94 | 132.41 |
| **7** | 5.10 | 110.29 | 112.85 | 117.95 | 123.05 | 128.16 | 133.26 | 135.81 |
| **7.5** | 5.27 | 112.66 | 115.29 | 120.56 | 125.83 | 131.10 | 136.38 | 139.01 |
| **8** | 5.28 | 115.49 | 118.13 | 123.41 | 128.69 | 133.97 | 139.26 | 141.90 |
| **8.5** | 5.48 | 117.99 | 120.73 | 126.21 | 131.69 | 137.16 | 142.64 | 145.38 |
| **9** | 5.39 | 120.77 | 123.47 | 128.86 | 134.25 | 139.64 | 145.03 | 147.73 |
| **9.5** | 5.70 | 122.55 | 125.40 | 131.11 | 136.81 | 142.51 | 148.22 | 151.07 |
| **10** | 5.87 | 124.65 | 127.59 | 133.46 | 139.33 | 145.20 | 151.07 | 154.01 |
| **10.5** | 6.28 | 126.31 | 129.45 | 135.73 | 142.01 | 148.29 | 154.57 | 157.71 |
| **11** | 6.10 | 129.48 | 132.53 | 138.63 | 144.74 | 150.84 | 156.94 | 160.00 |
| **11.5** | 6.75 | 130.53 | 133.90 | 140.65 | 147.40 | 154.15 | 160.90 | 164.27 |
| **12** | 7.13 | 133.06 | 136.63 | 143.75 | 150.88 | 158.01 | 165.13 | 168.70 |
| **12.5** | 7.45 | 135.25 | 138.98 | 146.43 | 153.88 | 161.33 | 168.78 | 172.51 |
| **13** | 7.71 | 138.01 | 141.86 | 149.57 | 157.28 | 164.98 | 172.69 | 176.55 |
| **13.5** | 7.78 | 141.60 | 145.49 | 153.27 | 161.05 | 168.83 | 176.61 | 180.50 |
| **14** | 8.02 | 144.56 | 148.57 | 156.58 | 164.60 | 172.62 | 180.63 | 184.64 |
| **14.5** | 7.33 | 149.18 | 152.84 | 160.17 | 167.50 | 174.83 | 182.15 | 185.82 |
| **15** | 7.15 | 151.93 | 155.51 | 162.66 | 169.81 | 176.97 | 184.12 | 187.69 |
| **15.5** | 6.82 | 154.91 | 158.32 | 165.13 | 171.95 | 178.77 | 185.58 | 188.99 |
| **16** | 6.57 | 156.54 | 159.82 | 166.40 | 172.97 | 179.54 | 186.11 | 189.40 |
| **16.5** | 6.46 | 158.00 | 161.23 | 167.69 | 174.15 | 180.62 | 187.08 | 190.31 |
| **17** | 6.41 | 159.11 | 162.31 | 168.72 | 175.13 | 181.54 | 187.95 | 191.16 |
| **17.5** | 6.09 | 160.36 | 163.41 | 169.50 | 175.59 | 181.69 | 187.78 | 190.82 |
| **18** | 5.84 | 161.29 | 164.21 | 170.06 | 175.90 | 181.75 | 187.59 | 190.51 |

D

| **Age (years)** | **SD** | **-2.5** | **-2** | **-1** | **0** | **+1** | **+2** | **+2.5** |
| --- | --- | --- | --- | --- | --- | --- | --- | --- |
| **0** | 1.91 | 44.81 | 45.77 | 47.69 | 49.60 | 51.51 | 53.43 | 54.38 |
| **0.25** | 2.81 | 52.04 | 53.44 | 56.25 | 59.06 | 61.87 | 64.68 | 66.09 |
| **0.5** | 2.73 | 58.27 | 59.64 | 62.37 | 65.10 | 67.83 | 70.56 | 71.93 |
| **1** | 2.67 | 67.44 | 68.77 | 71.43 | 74.10 | 76.76 | 79.43 | 80.76 |
| **1.5** | 3.00 | 73.36 | 74.86 | 77.86 | 80.85 | 83.85 | 86.84 | 88.34 |
| **2** | 3.19 | 79.03 | 80.62 | 83.81 | 87.01 | 90.20 | 93.39 | 94.99 |
| **2.5** | 3.55 | 82.27 | 84.05 | 87.60 | 91.16 | 94.71 | 98.26 | 100.04 |
| **3** | 3.76 | 86.01 | 87.88 | 91.64 | 95.40 | 99.16 | 102.92 | 104.80 |
| **3.5** | 3.81 | 89.45 | 91.36 | 95.17 | 98.99 | 102.80 | 106.61 | 108.52 |
| **4** | 4.14 | 92.54 | 94.61 | 98.76 | 102.90 | 107.04 | 111.18 | 113.25 |
| **4.5** | 4.13 | 96.12 | 98.18 | 102.32 | 106.45 | 110.58 | 114.71 | 116.78 |
| **5** | 4.49 | 98.62 | 100.87 | 105.36 | 109.85 | 114.34 | 118.83 | 121.07 |
| **5.5** | 4.58 | 101.48 | 103.77 | 108.35 | 112.92 | 117.50 | 122.07 | 124.36 |
| **6** | 4.74 | 104.33 | 106.70 | 111.43 | 116.17 | 120.90 | 125.64 | 128.01 |
| **6.5** | 4.91 | 106.93 | 109.39 | 114.31 | 119.22 | 124.13 | 129.05 | 131.51 |
| **7** | 4.81 | 109.85 | 112.26 | 117.07 | 121.88 | 126.69 | 131.51 | 133.91 |
| **7.5** | 5.13 | 112.23 | 114.80 | 119.93 | 125.05 | 130.18 | 135.31 | 137.87 |
| **8** | 5.27 | 114.61 | 117.25 | 122.52 | 127.79 | 133.07 | 138.34 | 140.98 |
| **8.5** | 5.42 | 117.24 | 119.95 | 125.38 | 130.80 | 136.22 | 141.64 | 144.35 |
| **9** | 5.71 | 119.27 | 122.12 | 127.83 | 133.54 | 139.25 | 144.96 | 147.81 |
| **9.5** | 5.97 | 121.44 | 124.43 | 130.39 | 136.36 | 142.32 | 148.29 | 151.27 |
| **10** | 6.25 | 123.47 | 126.60 | 132.85 | 139.10 | 145.35 | 151.60 | 154.72 |
| **10.5** | 6.36 | 126.73 | 129.91 | 136.27 | 142.64 | 149.00 | 155.36 | 158.55 |
| **11** | 6.51 | 129.37 | 132.62 | 139.13 | 145.64 | 152.15 | 158.67 | 161.92 |
| **11.5** | 6.39 | 132.93 | 136.13 | 142.52 | 148.92 | 155.31 | 161.70 | 164.90 |
| **12** | 6.43 | 135.59 | 138.80 | 145.23 | 151.65 | 158.08 | 164.50 | 167.72 |
| **12.5** | 6.39 | 138.48 | 141.67 | 148.06 | 154.44 | 160.83 | 167.21 | 170.41 |
| **13** | 5.97 | 141.81 | 144.80 | 150.77 | 156.74 | 162.71 | 168.69 | 171.67 |
| **13.5** | 5.99 | 143.41 | 146.40 | 152.38 | 158.37 | 164.36 | 170.34 | 173.34 |
| **14** | 6.00 | 144.81 | 147.81 | 153.81 | 159.81 | 165.81 | 171.80 | 174.80 |
| **14.5** | 5.82 | 146.06 | 148.97 | 154.78 | 160.60 | 166.42 | 172.23 | 175.14 |
| **15** | 5.97 | 146.47 | 149.45 | 155.42 | 161.39 | 167.36 | 173.33 | 176.32 |
| **15.5** | 5.83 | 147.40 | 150.31 | 156.14 | 161.97 | 167.80 | 173.63 | 176.55 |
| **16** | 6.04 | 147.13 | 150.14 | 156.18 | 162.21 | 168.25 | 174.28 | 177.30 |
| **16.5** | 5.88 | 147.77 | 150.72 | 156.60 | 162.48 | 168.36 | 174.25 | 177.19 |
| **17** | 5.76 | 148.31 | 151.19 | 156.94 | 162.70 | 168.45 | 174.209 | 177.09 |
| **17.5** | 5.77 | 148.35 | 151.23 | 157.00 | 162.77 | 168.54 | 174.3058 | 177.19 |
| **18** | 5.76 | 148.45 | 151.33 | 157.08 | 162.84 | 168.60 | 174.3513 | 177.23 |
